# Methods for Authenticating Participants in Fully Web-Based Mobile App Trials from the iReach Project: Cross-sectional Study

**DOI:** 10.2196/28232

**Published:** 2021-08-31

**Authors:** Jodie L Guest, Elizabeth Adam, Iaah L Lucas, Cristian J Chandler, Rebecca Filipowicz, Nicole Luisi, Laura Gravens, Kingsley Leung, Tanaka Chavanduka, Erin E Bonar, Jose A Bauermeister, Rob Stephenson, Patrick S Sullivan

**Affiliations:** 1 Department of Epidemiology, Rollins School of Public Health Emory University Atlanta, GA United States; 2 School of Medicine Emory University Atlanta, GA United States; 3 Center for Sexuality and Health Disparities University of Michigan Ann Arbor, MI United States; 4 Department of Psychiatry and Addiction Center University of Michigan Ann Arbor, MI United States; 5 Injury Prevention Center University of Michigan Ann Arbor, MI United States; 6 School of Nursing University of Pennsylvania Philadelphia, PA United States; 7 Department of Systems, Population and Leadership School of Nursing University of Michigan Ann Arbor, MI United States

**Keywords:** HIV, mHealth, recruitment, fraud, adolescent MSM, prevention, MSM, RCT, enrollment, data authentication, data quality, methods, participants

## Abstract

**Background:**

Mobile health apps are important interventions that increase the scale and reach of prevention services, including HIV testing and prevention counseling, pre-exposure prophylaxis, condom distribution, and education, of which all are required to decrease HIV incidence rates. The use of these web-based apps as well as fully web-based intervention trials can be challenged by the need to remove fraudulent or duplicate entries and authenticate unique trial participants before randomization to protect the integrity of the sample and trial results. It is critical to ensure that the data collected through this modality are valid and reliable.

**Objective:**

The aim of this study is to discuss the electronic and manual authentication strategies for the iReach randomized controlled trial that were used to monitor and prevent fraudulent enrollment.

**Methods:**

iReach is a randomized controlled trial that focused on same-sex attracted, cisgender males (people assigned male at birth who identify as men) aged 13-18 years in the United States and on enrolling people of color and those in rural communities. The data were evaluated by identifying possible duplications in enrollment, identifying potentially fraudulent or ineligible participants through inconsistencies in the data collected at screening and survey data, and reviewing baseline completion times to avoid enrolling bots and those who did not complete the baseline questionnaire. Electronic systems flagged questionable enrollment. Additional manual reviews included the verification of age, IP addresses, email addresses, social media accounts, and completion times for surveys.

**Results:**

The electronic and manual strategies, including the integration of social media profiles, resulted in the identification and prevention of 624 cases of potential fraudulent, duplicative, or ineligible enrollment. A total of 79% (493/624) of the potentially fraudulent or ineligible cases were identified through electronic strategies, thereby reducing the burden of manual authentication for most cases. A case study with a scenario, resolution, and authentication strategy response was included.

**Conclusions:**

As web-based trials are becoming more common, methods for handling suspicious enrollments that compromise data quality have become increasingly important for inclusion in protocols.

**International Registered Report Identifier (IRRID):**

RR2-10.2196/10174

## Introduction

### Background

Web-based trial recruitment, enrollment, and data collection are increasingly common in research, particularly those focused on the use of mobile health (mHealth) apps. The benefits of using web-based methods include faster and cheaper recruitment, particularly in rural areas [1]. Traditional in-person recruitment strategies are more complicated with adolescent men who have sex with men as they are not able to frequent bars and may not attend gay pride events and other common locations for recruitment in studies of men who have sex with men. This, coupled with the common use of social media apps in this age group, makes web-based recruiting efficient and more generalizable. mHealth apps hold promise to increase the provision of prevention services [2,3] and to reach populations such as adolescent men who have sex with men and rural men who have sex with men who may face interpersonal and structural barriers to seeking in-person prevention services [4-6]. These apps and the use of web-based study methods can be particularly useful for adolescent men who have sex with men residing in rural communities who may not have shared their sexuality with their family and friends and where access to services is challenging. The distribution of these apps through trials is more widespread on the internet, and these apps increase the scale and reach of prevention services, including HIV testing and prevention counseling, pre-exposure prophylaxis, condom distribution, and education, all of which are required to decrease incidence rates [7]. However, web-based trials increase the need for careful scrutiny of forms of fraudulent activity both as an issue of data quality (ie, multiple entries and ineligible participants providing inaccurate age to participate) and as an issue of protection for the adolescent men who have sex with men enrolling in the study. It is critical to ensure that the data collected through this modality are valid and reliable [8].

Authentication for fully web-based studies requires a multimodal approach of electronic and manual verification that may require substantial effort compared with traditional in-person studies [9-11]. There are many examples in the literature on the frequency of fraud in web-based studies. In 2019, Ballard et al [12] categorized 28.7% of their web-based surveys as fraudulent and another 10.1% as potentially fraudulent. In addition, a fully web-based youth-specific HIV study in 2008 identified 675 persons suspected of fraudulent enrollment through multimodal processes [9], and an adolescent men who have sex with men–specific survey published in 2013 found 559 fraudulent cases [13]. Two analyses in 2020 examining issues with web-based recruitment in men who have sex with men concluded that fraud was common, that manual methods work but are resource intensive, and that additional research should be completed to find affordable methods to limit fraudulent enrollment in studies [14,15].

### Objectives

Trials of mHealth tools for HIV prevention pose unique challenges, including the authentication of potential participants and the prevention of fraudulent attempts to enroll in studies [16,17]. This study describes the authentication and fraud prevention protocols used in the iReach project, a randomized controlled trial (RCT) of a multilevel life skills intervention that uses mobile apps to reduce vulnerability among men adolescent men who have sex with men [18]. We present the multistep validation process for this web-based adolescent trial, which included electronic programmed comparisons; the use of a manual checklist; and fraud detection methods, including social media. We describe the application of these steps in the trial and provide examples and metrics for the more common types of fraudulent activities, including a brief case scenario for illustration. The strategies used could benefit others who are working on recruitment and enrollment in web-based studies.

## Methods

### iReach Trial Methods

Methods for conducting the ongoing iReach trial have been described elsewhere [18]. In brief, the trial aimed to explore the efficacy of a multilevel life skills intervention delivered through a web app to 499 adolescents (aged 13-18 years), same-sex attracted, cisgender males (people assigned male at birth who were identified as men) in four US regions, and an additional 101 adolescent men who have sex with men nationally. The participants were a racially and ethnically diverse sample with at least 50% (300/600) identity as people of color or from rural communities. After enrollment, eligible participants assigned to the experimental arm had access to the iReach web app over 12 months of the study. Within the web app, they had access to activity-based life skills modules across 14 key life areas, set goals, monitor progress toward these goals, work on these goals using the peer mentor video chat feature, and access to a locator feature to find community resources that are welcoming to lesbian, gay, bisexual, transgender, queer, questioning, and other sexual and gender minority individuals to support a healthy life and achieve their goals. Participants randomized to the control arm of the trial had access to the locator feature of the intervention due to adolescent men who have sex with men’s vulnerability to HIV and sexually transmitted infections. At the end of the 12-month period, participants in the control arm were given full access to the iReach web app for 3 additional months. The primary outcomes of the study were cognitive factors linked to the ability to use HIV prevention and behavioral intentions to use HIV prevention. Participants received a US $30 Amazon gift card for surveys at baseline and 12-month follow-up; control participants received an additional US $30 gift card for a 15-month follow-up. Participants received a US $25 Amazon gift card for the 3-, 6-, and 9-month follow-up surveys. The University of Pennsylvania Institutional Review Board (IRB) served as the IRB of the record for this study. In accordance with the National Institutes of Health Common Rule, the University of Michigan and Emory University IRBs entered IRB authorization agreements with the University of Pennsylvania IRB. A waiver of parental consent was obtained for minor participants under the age of 18 years.

### Recruitment

Recruitment was primarily completed through demographically targeted banner advertisements on social media platforms (ie, Snapchat and Facebook). Additional community engagement using print and media advertisements supplemented the social media advertisements with a link to screening eligibility on the web. All recruitment advertisements asked young men to help test a new health app and included photos of racially and ethnically diverse young men and a link to follow for eligibility.

Interested participants who clicked on the banner ads or accessed the screening survey link were screened for eligibility on the web and completed an electronic informed consent process [19].

### Authentication Strategies

#### Automatic Authentication Strategies

A series of automated authentication strategies identified potentially ineligible participants.

#### Phone and Contact Information Verification

Potential participants were required to submit their mobile phone number on the screener survey to receive a 3-digit verification code, which they received through SMS text messages. After receiving the 3-digit code, potential participants would then enter the screener survey to validate their mobile phone number for the study.

Participants who failed to input the code during screening were not able to continue the screening survey. When a participant entered an incorrect code, the study team was notified, and the participant was offered assistance in receiving and inputting the code. After verification of the 3-digit code, participants submitted the required information (preferred name, email address) and optional additional contact information (home address, social media handles). These data were part of the manual checklist verification and were used throughout the study for ongoing analysis of enrollment and survey data that would trigger additional reviews.

Those who passed the eligibility criteria, verified their mobile phone number, provided contact information that included their zip code, and consented to the study procedures were routed to the baseline questionnaire. Both automatic and manual verification processes were completed on an ongoing basis for new potential participants three times a week. After full verification, the eligible participants were randomized according to the study protocol.

##### Questionnaire Data Evaluation

###### Evaluation Methods and Justification

The screening and baseline questionnaire data were evaluated in the following three ways: (1) by identifying possible duplication of contact information with data from previously registered participants, the team ensured that the participants were new and unique; (2) by identifying inconsistencies in data collected at screening and survey data, the team could identify potentially fraudulent or ineligible participants (those who were outside the eligible age group, who did not report same-sex attraction, who reported being HIV positive at baseline, or did not reside in the targeted recruitment areas were deemed ineligible); and (3) by reviewing baseline completion times, the team avoided enrolling participants or bots who did not complete the baseline questionnaire.

###### Duplication Checks

Duplication checks were initiated for all participants using a baseline questionnaire record. SAS programs were run to check the newly submitted record against all previous baseline questionnaires to check for duplicates of email addresses, mobile numbers, IP addresses, mailing addresses, social media handles, and preferred names. Potential matches identified by the automated checks were manually reviewed by the study staff. If a potential participant had already been evaluated for study enrollment or had submitted multiple screening surveys with inconsistent information, the participant was not passed to the next step (manual verification). Similarly, the electronic participant management system, Study Management and Retention Toolkit (SMART; developed by Emory University Center for AIDS Research), which was used to track, manage, and contact study participants, searches for exact and partial matches in contact information for each new participant in the SMART system.

###### Data Comparisons

SAS programs compared the screening survey and baseline questionnaire data for each participant to identify conflicting or inconsistent information between the two surveys using the data elements collected.

Age (in years) was collected in the screening survey, and date of birth (DOB) was collected from the baseline questionnaire. If the age of the participant reported in the screener did not match the age calculated from their reported DOB and baseline questionnaire completion date, these records were flagged for manual review.

IP addresses can identify unique users and their locations. Although IP addresses may be transient, can be duplicated due to institutional IP addresses, or can be changed using proxy servers [11], inconsistencies between the location of the IP address and the self-reported address of participants were considered important indications of potentially fraudulent enrollment. An automated program compared the state of the mailing address submitted by the participant and the state recorded through the IP address. In addition, because eligible participants completing the screening survey were immediately referred to the baseline questionnaire, a second program compared the IP address locations of screening survey completion and baseline questionnaire completion.

###### Baseline Questionnaire Completion Time

The baseline questionnaire was designed to take approximately 30 minutes. Baseline questionnaires completed in less than 20 minutes were flagged for manual review.

Each participant’s baseline record was assigned a status (complete, partial, or duplicate) and a processing date. Reports of completion scores were generated as participants attempted to enroll in the study; therefore, patterns of multiple fraudulent attempts emerged over time and could be readily identified, and potentially fraudulent participants prevented from enrolling in the study.

###### Baseline Questionnaire Completion Scores

Participants had the option to skip specific survey questions that they preferred not to answer. To ensure that the enrolled participants were meaningfully engaged in the survey, a random subset of baseline questions that were not impacted by skip patterns was assessed for completion. Participants who completed less than 60% (17/27) of the subset of questions were not referred for enrollment to exclude potential fraud from bots and those who completed research surveys for profit [20]. Similarly, a subsample of the primary outcome questions from the baseline questionnaire was assessed for completion. Participants who completed less than 70% (44/62) of these primary outcome questions were not referred for enrollment. The lack of completion at this early stage may forecast challenges in obtaining complete outcome data within this study, as participants with low completion rates may not understand, recall, or be able to provide judgment on items in the format requested. Participants who surpassed the 60% (17/27) and 70% (44/62) thresholds for the subset of questions and the primary outcome questions, respectively, but completed less than 80% (22/27 and 50/62, respectively) of either question set were flagged for manual review.

#### Manual Authentication Strategies

##### Manual Review Process

After the automated authentication checks, a manual review was conducted for participants who flagged for additional review. This manual validation used a checklist (Multimedia Appendix 1), and case report forms were developed to monitor and document the process. A manual review of participants was completed in 1 to 2 business days.

##### Assessment of Flagged Data

Research staff manually checked all responses of age, IP address comparisons, completion scores, and time stamps, and documented assessments of explainable inconsistencies in the electronic case report forms (eg, a participant identified as 17 years old but added a DOB that was 2 weeks in the future).

##### Survey Review

If participants had baseline questionnaire completion proportions near the threshold (17/27, 60% for random assortment and 44/62, 70% for primary outcomes), or had multiple flags for review, a manual review of the survey questionnaire was performed to search for patterns of illogical answers (eg, conflicting answers or the same response for all questions or a *Christmas tree* pattern of answering questions, as previously suggested in the literature) [11]. If a pattern was found, it was documented on the manual enrollment checklist, and the participant was not enrolled.

##### Social Media Review

Although not required for participation, all adolescent men who have sex with men enrolled were asked to submit their social media handles to the study. Of those who provided contact information, 65.18% (863/1324) provided their social media handles. As part of the consent process, participants were asked their permission for the study staff to use these social media platforms to contact the participant or verify their information. The social media review was designed to supplement the enrollment process to verify demographic information (eg, age and gender), the location of the participant, and other helpful information if publicly available on the profile (eg, email address).

##### SMART Enrollment

The SMART participant management system contains a GPS-specified search engine that allows for the automatic population of zip codes when an address is entered. If the automatically indexed zip code did not match the zip code submitted by the potential participant, this triggered a review for similar addresses and exploration of the address manually. This information was noted in the manual enrollment checklist.

##### Email Verification

Once all other checks were completed and a determination was made that there was no irregular activity, participants completed an email verification. Participants had 30 days from the issuance of the email verification attempt to respond to the study team. Participants who did not complete this step were not enrolled in the study. Weekly reminders were sent to the participants to increase the likelihood of completing this step.

#### Ongoing Quality Assurance

##### Age Verification

iReach collected data every 3 months during the follow-up surveys. The calculated ages from the follow-up surveys were compared with the baseline questionnaire. Variations were manually reviewed, and a determination was made by the study team if participation was discontinued because of concerns about age verification.

##### Alternate Phone and Email Comparisons

At the time of each follow-up survey, participants were asked to provide additional phone numbers or email addresses as alternatives if the study team could not reach them using their primary contact information. Periodically, the study team evaluated possible matches between each of the alternative contact information provided and the contact information from other participants’ main phone numbers and email addresses as a form of potential fraud prevention. If exact or partially matching contact information was found, the two participants were flagged and reviewed to determine whether they were duplicate or dually enrolled.

##### Other Administrative Study Discontinuations

Participants were also removed from the study for any of the following reasons: (1) failure to make contact after multiple engagement attempts, (2) voluntary withdrawal by the participant, (3) errors in the enrollment of the participant that could not be corrected, (4) incarceration of the participant, (5) report of the participant being deceased, or (6) in the event of other unanticipated events that precluded further study participation.

## Results

### Research Findings

**Figure 1:**
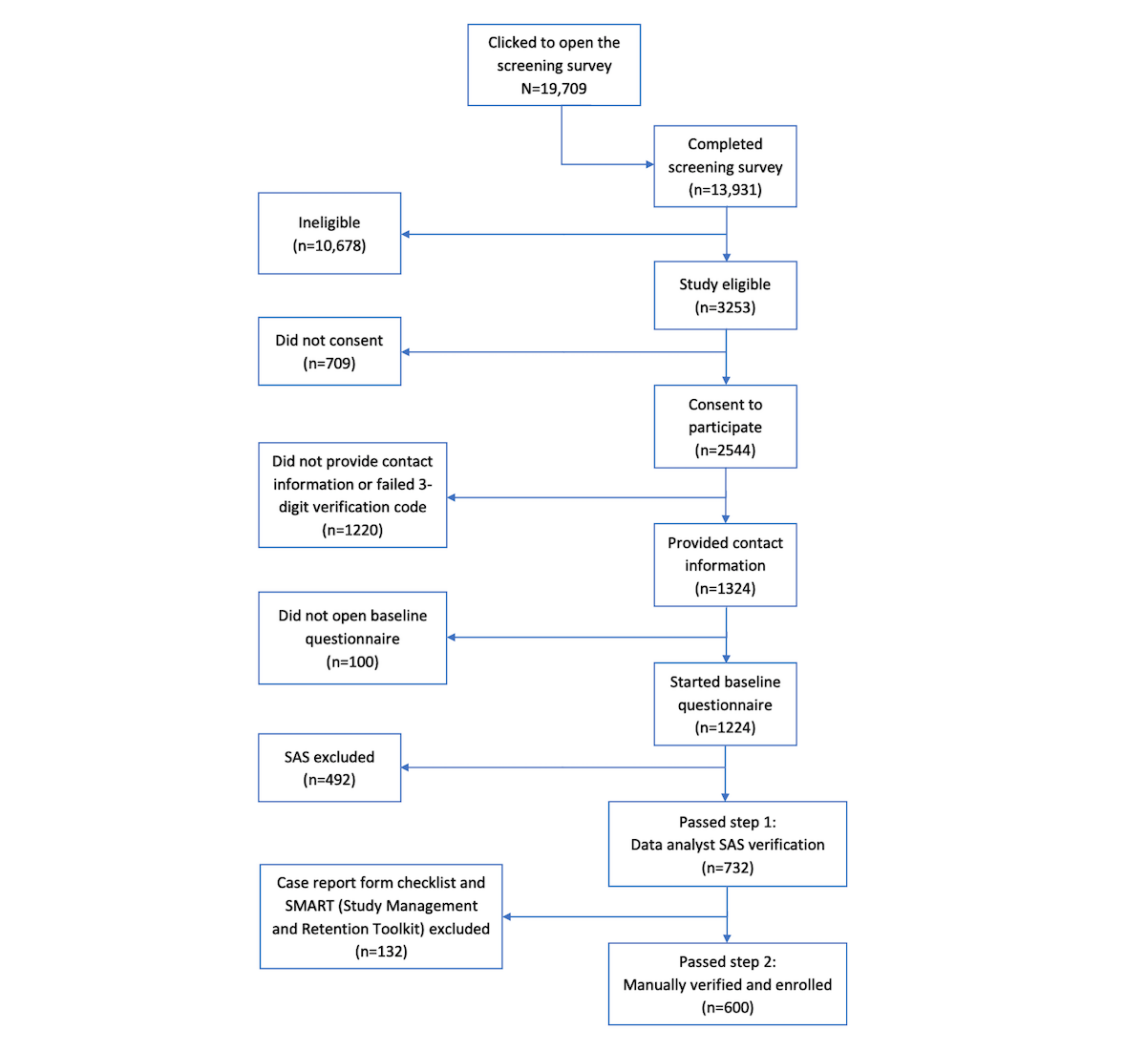
Flow diagram of iReach participant enrollment.

Of the 19,709 visitors to the iReach project screening survey, 13,931 (70.68%) completed the screening survey. Of these, 23.35% (3253/13,931) were eligible for the study. Of the 3253 visitors who were deemed eligible, 2544 (78.2%) consented to participate in the study. After excluding potential participants who did not provide contact information or who failed the 3-digit phone verification, 92.44% (1224/1324) started the baseline questionnaire. Figure 1 shows a flow diagram of enrollment based on the responses of potential participants.

Upon completion of the baseline questionnaire, responses were verified by automatic electronic authentication using the SAS program. This automatic authentication was completed for potential participants in bulk every Monday, Wednesday, and Friday. Table 1 demonstrates that although 1224 participants started the baseline questionnaire, 492 were excluded because of SAS-programmed automatic authentication failures, most of which (n=252) excluded at this stage were excluded because they did not complete 60% (17/27) of the random subset of questions or 70% (44/62) of primary outcome questions. As iReach is a regionally bound RCT for HIV seronegative youth, IP addresses outside the United States were excluded from moving forward (n=8). In addition, several duplications of participants (n=177) who already existed within the study or the SMART participant management system were excluded. The remaining participants were referred for manual verification.

**Table 1:** Participant status resulting from automated authentication (N=1224; started baseline).

Status	Participants, n (%)
Failed SAS eligibility recheck^a^	32 (2.61)
Duplication or already enrolled	177 (14.46)
IP address outside the United States	8 (0.65)
Failed completion score requirements^b^	252 (20.59)
Case study participant attempts to enroll	23 (1.88)
Passed, referred to manual authentication	732 (59.8)

^a^Did not meet race criteria: n=30; did not meet HIV status criteria: n=2.

^b^<70% of primary outcome questions or <60% of the random subset of questions.

Tables 2 and 3 show the verification and failure of potential participants referred for manual authentication, respectively. The manual review process was used to exclude 132 individuals (Multimedia Appendix 1). As several areas of data were assessed for irregularities before exclusion, the causes for exclusion in this stage were not mutually exclusive but are noted in Table 3.

**Table 2:** Participant status resulting from manual authentication (n=732; referred).

Status	Participants, n (%)
Failed, no checklist completed (duplicate)	9 (1.2)
Failed manual checklist and removed	123 (16.8)
Passed and enrolled	600 (81.9)

**Table 3:** Manual authentication failure reasons for potential participants who were removed from study enrollment (n=123).

Reasons failed^a^	Participants, n (%)
Time stamp fail	33 (26.8)
Age comparison screener and baseline fail	20 (16.3)
Duplicate check fail	28 (22.8)
Suspicious pattern survey response fail	33 (26.8)
Social media check fail (if provided)	20 (16.3)

^a^Failure to move on to enrollment was based on the manual review checklist. The numbers reported are not mutually exclusive.

A total of 132 potential participants failed manual authentication during manual checklist completion. The reasons for failure during manual authentication varied and could overlap if potential participants had multiple issues with the data they provided. The most common reasons for manual authentication failure were a time stamp failure (n=33) or suspicious survey response patterns (n=33). A time stamp failure occurred when potential participants had unusually short or unusually long completion times for their screener survey or baseline questionnaire. A suspicious survey response pattern occurred when the potential participant provided conflicting responses or responded in a pattern (eg, selecting the same response for every answer). Potential participants were also excluded if manual authentication revealed that they did not meet eligibility age requirements for the study, if their social media profile or profiles (if provided) revealed that the potential participant was not who they said they were (ie, did not meet eligibility requirements for age, gender, and location), or if it was discovered that they were duplicates who managed to pass through the automatic authentication process. One specific cluster of 23 potentially fraudulent enrollments was highlighted in the case study provided.

### Case Study: The Wisteria Participant

While manually verifying a potential participant for the iReach project, a study team member noted that the zip code provided by the potential participant did not match the SMART-derived zip code (note that the street name has been changed). This flagged an electronic review of the participant data that found the address provided was a partial match with an already-enrolled participant and differed only by house number. In addition, the contact phone number provided by the potential participant was the secondary phone number of the currently enrolled participants with the same street name.

A study team member attempted to contact the potential participant to ask for additional verification to ensure that the potential participant could be authenticated. When the potential participant did not respond after multiple attempts at contact, they were not enrolled in the study. The previously enrolled participants with a similar address and phone number were sent a message that attempts to duplicate enrollment would be grounds for dismissal from the study.

Twenty-three additional attempts to enroll in the study came from different house numbers on Wisteria Street. Owing to the electronic and manual verification systems in place in the study, multiple attempts for duplication of enrollment were identified and prevented. The enrolled participants were contacted and advised that they were being removed from the study. This example demonstrates the effectiveness of using electronic strategies that include both exact matches and partial matches for addresses to discover potential fraud cases.

## Discussion

### Principal Findings

This study examines methods for authenticating unique participants for the iReach project, a fully web-based RCT multilevel skills intervention for HIV prevention with adolescents, a population that is easier to enroll on the web. Using a multimodal approach to authentication, 624 potential participants were excluded from enrollment, including those who attempted to enroll more than once. Most participants were excluded by automated data reviews, with a smaller number requiring manual authentication by the study staff.

Over the past 15 years, researchers have acknowledged the threat that potentially fraudulent cases can have on the internal and external validity of their trials [8,9,17]. Several of the authentication checks used in the iReach project mirror those of previous studies, including the electronic verification strategies of IP addresses, the comparison of demographic information at multiple time points (ie, age), and survey time stamp review. Similar to other human verification strategies, manual reviews that look for patterns of illogical responses and requests for the submission of additional proof of eligibility were also included in iReach. The iReach project has also extended previously reported verification processes by improving the methods of possible fraud detection, particularly through the use of electronic authentication strategies. By using automated systems to identify exact and partial matches of demographic information (eg, name and email address) and the SMART participant management system to verify addresses based on GPS locations, many possibly fraudulent cases were eliminated before manual authentication approaches were engaged. Furthermore, the completion thresholds used for all questions and primary outcome questions—60% (17/27) and 70% (44/62), respectively—reduced the number of possibly fraudulent cases that underwent manual authentication. An additional highlight of the iReach project approach was the use of social media for verification. Given that estimates of social media use among youth are as high as 97% [21], using social media data can increase the sensitivity of detecting possible fraudulent enrollments, and some researchers have gone a step further by asking participants to provide a current selfie to match to social media profiles, as described by Bonar et al [22]. Although not a requirement for this study, most participants provided a social media profile. Finally, the use of a single report provided to the study team that detailed possible inaccuracies offered an efficient checklist to ensure a systematic approach to manual authentication.

### Strengths and Limitations

It is important to consider how best to characterize the sensitivity and specificity of fraud detection systems. Enrolling fraudulent participants introduces bias into the data, and the detection consumes resources. When working with youth, authentication can limit the potential harm of youth inadvertently interacting with fraudulent accounts or nonminors. However, there is a balance needed in fraud detection, as we strive to include a diversity of participants in the studies. Automated electronic fraud detection methods have the potential to introduce selection bias, as it is plausible that residents of high-density housing developments are at higher risk of being classified as potentially fraudulent based on the similarity of addresses or shared IP addresses than residents of single-family dwellings. This could also be true for participants in college, given that this study included those aged 18 years. A fraud detection system that is only automated might make 1 determination, whereas a fraud detection system that uses both automated and manual verification will be more likely to uncover the reasons for the similarities. Furthermore, 22.8% (28/123) of potential participants who failed manual authentication were discovered to be duplicates, even though they had avoided detection during the initial automatic authentication process, which demonstrates the importance of a combination of both automated and manual authentication for data quality. Potential iReach participants whose data indicated a need for manual review were not automatically or always excluded due to the manual check if staff found explanations for the issue that triggered the manual review. This included participants with long completion times for the baseline questionnaire or those with an IP address that was different from their state address, often due to travel or moving. Although manual reviews certainly take longer and are more time consuming for staff, the targeted use of manual review triggered by the automated review of data resolved issues and inconsistencies by contacting participants. As suggested by Bauermeister et al [23] and Ballard et al [12], this focused manual review allows opportunities to resolve more nuanced situations and improve the specificity of the fraud detection algorithm and keep these participants in the study if the manual review is passed. Whenever possible, study teams strive to strike a balance between automatic and manual authentication strategies to produce the highest quality of research.

### Conclusions

Research teams recruiting on the web should be vigilant to maintain scientific rigor in the methods of recruitment and retention. As technology continues to advance, researchers should periodically update methods to ensure the authenticity and uniqueness of participants. Reviews of the design and implementation of electronic and manual strategies for authentication should be performed periodically to ensure that the validity of the study sample is maintained. The careful construction of ways to avert fraud in the design stages can help prepare research teams for unanticipated challenges within this environment, save time and money with detection efforts, and preserve data quality.

## Appendix

Multimedia Appendix 1 iReach enrollment checklist.

## Abbreviations

DOB: date of birth
IRB: Institutional Review Board
mHealth: mobile health
RCT: randomized controlled trial
SMART: Study Management and Retention Toolkit
