# Supplementary material for: Methods for Authenticating Participants in Fully Web-Based Mobile App Trials from the iReach Project: Cross-sectional Study
Source: JMIR Mhealth Uhealth. 2021 Aug 31;9(8):e28232. doi: 10.2196/28232 (PMC8441600; doi:10.2196/28232)
Supplement: Multimedia Appendix 1 [file mhealth_v9i8e28232_app1.docx]

DataFax # 042 Plate # 001 Visit # 001 iREACH

# Enrollment Checklist Page 1 of 2

Instructions: *Complete this form in iDataFax after receiving the list of Participants to Validate. Notify data analyst once the Enrollment Checklist has been completed for new participants. After data checkpoint, complete Ready-to-Randomize and Randomization Forms in iDataFax and the CHCR Web Form in iReach Dashboard.*

DataFax ID:

| Staff Initials: |  |  |  |
| --- | --- | --- | --- |

Screening ID: Date Completed:

*Month Day Year*

|  |  |  |
| --- | --- | --- |

Participant Code:

**Section 1. Staff Validation of Potential Participant**

| **Step #1: Time-stamp review**  Step completed?  Yes  No  Passed the step?  Yes  No |  | Notes: | |
| --- | --- | --- | --- |
| **Step #2: Age comparison**  Step completed?  Yes  No  Passed the step?  Yes  No |  | Notes: | |
| **Step #3: Duplicate enrollment check**  Step completed?  Yes  No  Passed the step?  Yes  No | | | Notes: |
| **Step #4: Review survey for suspicious response patterns**  Step completed?  Yes  No  Passed the step?  Yes  No | | | Notes: |
| **Step #5: Cross-check email with social media profile information**  Step completed?  Yes  No  Passed the step?  Yes  No | | | Notes: |

DataFax # 042 Plate # 002 Visit # 001

iREACH

# Enrollment Checklist Page 2 of 2

Instructions: *Complete this form in iDataFax after receiving the list of Participants to Validate. Notify data analyst once the Enrollment Checklist has been completed for new participants. After data checkpoint, complete Ready-to-Randomize and Randomization Forms in iDataFax and the CHCR Web Form in iReach Dashboard.*

DataFax ID:

|  |  |  |
| --- | --- | --- |

Screening ID:

Participant Code:

**Section 1, continued. Staff Validation of Potential Participant**

| **Step #6: Email verification** Notes:  Step completed?  Passed the step?  Yes  Yes  No  No  Date participant responded:  *Month*  *Day*  *Year* |
| --- |
| **Step #7: Three-digit code authentication** Notes:  Step completed?  Yes  No  Passed the step?  Yes  No |

**Section 2. Results of Validation**

1. Based on steps above, is person eligible for enrollment and can move on to data checkpoint?

No

a. If no, explain additional steps to validate participant:

Yes

| *If eligible, update DataFax ID by adding a* ***1*** *to the last box.*  *If ineligible, update DataFax ID by adding a* ***9*** *to the last box.* | b. After additional steps completed, was person eligible for enrollment? |
| --- | --- |

Yes No

Form is ready for data checkpoint. Do not proceed to randomization until completed.
